# Questionnaire about psychology/disease correlation – II

**Published:** 2011-11-24

**Authors:** D Dragoş, DG Ojog, MD Tănăsescu

**Affiliations:** * "Carol Davila” University of Medicine Bucharest, 1st Internal Medicine Department, University Emergency Hospital Bucharest, Romania; ** "Carol Davila” University of Medicine Bucharest

**Keywords:** personality inventory, psychological profile, psychological predisposition to disease

## Abstract

**Objective.** To further evaluate the adequacy of the items in our questionnaire aimed at unraveling the possible correlations between psychological features and internal disorders. This paper is dedicated to the items exploring the individual’s interaction with other people.

**Method.** The items are divided into several subdomains. For each subdomain, we have calculated the correlations between the items of the respective subdomain (inner associations) and with the items in other subdomains (outer associations) by means of chi square test or Fisher exact test as dictated by statistical reasons. We examined the answers from our first 10192 respondents.

**Results and conclusions.** Many inter-item correlations are the consequence of higher or lesser degrees of synonymy. Those within a given subdomain confirm the adequate allocation of items. Those bridging different subdomains may point either to incorrect assignments, or to semantic inclusion relations. Other results are not explicable by semantic similarity, and probably reveal psychological subtleties, such as: most individuals have a sense of undeservedness when badly treated by other people; those easily hurt by insults and humiliations have a propensity to timidity and/or emotivity; the subjects who shun conflicts are more prone to persistent thoughts, brooding people are more sensitive and more prone to conflicts, injustice-indignant people frequently get into conflict although they declare to be bothered by dissent etc. But at the heart of all the PFs in the Interaction-with-other-people domain there seems to be the sense of being undervalued, which should probably be the key issue to be addressed by any therapeutic interventions for diseases psychoemotionally determined by disturbed interpersonal relationships.

**Abbreviations:** PF = psychological feature; Chisq = chi-square; OdRa = odds ratio; OdRaCL = odds ratio confidence limits; ErrProb = probability of error

## Introduction

In a previous paper [**1**], we have given the background and the reasons for conducting the present study, as well as the way we have built our questionnaires. Briefly, in the search for personality features that might predispose to certain symptoms/disorders, the general ones (anxiety, hostility, depression, etc.) prove to be unrewarding, as each of them appears to be correlated with several areas of pathology, therefore lacking specificity. Consequently, based on the results of a former retrospective study (some of whom we have presented in previous papers), we have conceived a triple questionnaire, that we are using in the present study.

### Aim

At this stage of our study, our goal is to establish to what degree the items in our questionnaires are representative for the psychological features we have ascribed them to and which of them are highly correlated with each other, with the future aim of conceiving an objective psychological evaluation system and a method for testing the consistency of each respondent’s answers.

## Methods

In a previous paper [**1**], we have presented the structure of our triple questionnaire (http://drdorindragos.ro/causesdiseases.html). Like that paper, this one will be concerned only with the results yielded by the first two questionnaires. We remind the reader that the items are divided into four main domains (“Preoccupation with the basal conditions of existence”, “Interaction with other people”, “Action, will/volition, self–assertion”, “Preoccupation with the exterior”), each with several subdomains, and some with several subsubdomains.

We have presented the results regarding the first domain in our previous paper. In the present one we are examining those concerning the second domain (“Interaction with other people”). Here are the subdomains pertaining to this second domain [in brackets is the shorthand for the subdomain’s name used throughout this paper]: a. Preoccupation with couple and/or (the relationship with) his/her partner [discontent/worry about couple/partner]; b. Preoccupation with the relationship with his/her relatives/friends [relatives/friends]; c. Preoccupation with social group [social–group]; d. Sociability, desire for company [sociability]; e. Tendency to isolation [tendency–to–isolation]; f. Timidity [timidity]; g. Emotivity [emotivity]; h. Tendency to hide one’s feelings/emotions [feelings–hiding]; i. Tendency to rumination (persistent thoughts) [rumination]; j. Desire to be appreciated [appreciation–desire]; k. Proneness to conflict [conflict–proneness]; l. Dislike for injustice, desire to be treated fairly [injustice–dislike]; m. Punctuality [punctuality]; n. Justice [justice–desire]; o. Frankness [frankness].

In our initial classification tendency–to–isolation, timidity, and emotivity subdomains were considered subsubdomains of a broader subdomain, called “socially avoiding nature”. Similarly, injustice–dislike, punctuality, justice–desire, and frankness subdomains were joined as subsubdomains of a larger subdomain, “desire for equity/fairness”. As these associations could actually be artificial, and, for the sake of simplicity, we decided to consider them as distinct subdomains.

We have estimated the correlation between each two different items using the chi square (χ²) test or Fisher exact test where appropriate (actually Fisher exact test was never necessary due to the strength of our sample). The respondents were asked to fill in the items by checking one out of five variants. However, for the sake of clearness, we have reduced the five variants to only two: below and above the average for the respective item.

Due to the large number of calculated parameters (614 × 613 ÷2 = 188191), we have considered as statistically significant only those results with an error probability (ErrProb) (evaluated by using the chi square test) of less than 10⁻⁷ = 0.0000001. Therefore, each of our results has a probability of at least 0.9999999 to be correct. Therefore, the probability for all of them to be correct is at least 0.9999999188191 = 0.98 (which is above the generally accepted cut–off point of 0.95). Actually, due to the large number of very significant correlations (almost 2000 with an ErrProb <10⁻³⁰) and to the limited space available for our article, we had to restrict our analysis to the most significant ones – therefore, all the results we present in this paper have a ErrProb of at least 10⁻¹⁰⁰, corresponding to a ErrProb <10⁻⁸⁵ for each calculated parameter.

We have performed this on our first 10192 respondents (1711 M, 8481 F), aged 33,98 ± 11,39 yrs (35,43 ± 12,52 yrs for males, 33,68 ± 11,13 yrs for females).

We should stress out that *some items deliberately express the opposite of their subdomain*. For these items we have taken into account the opposite of their scores in our calculations. For example, for the item “I like to be alone” in the sociability-subdomain, a score of “Little” as checked by the respondent was taken into account as a score of “Much” in our calculations.

## Results

There were 84 correlations with an ErrProb evaluated as zero (actually <10⁻³⁰⁷). Some of these correlations were among:

° (almost) synonymous items: ● I get excited/I am moved/I am touched – I am emotional; ● I suffered injustice – Life has been unfair to me; ● I try to arrive in time – I am punctual; ● I want company, I like being in the company of people – I like to receive visits as well as to pay visits etc.

° items semantically including one another: ● I was unjustly punished – Life has been unfair to me; ● It is important to have friends – I like being with my group of friends etc.

° obviously related items: ● I like parties because I have the opportunity to interact with other people – I like to have an intense social life etc.; ● When I am in the company of unknown people, I interact with them – I act naturally in the presence of strangers etc.

The less strong correlations seem to follow the same pattern, but among them we have found some less predictable associations, which may offer some insights into the human psychology. For the sake of brevity we shall restrict our presentations to those results. Actually we shall present only the associations among items pertaining to different subdomains, leaving outside the strong correlations among items in the same subdomain (which are nevertheless very useful in proving the right assignment of these items to their subdomain).

**Table 1:** **Appreciation–desire** correlated with **timidity**

The item to be correlated with	OdRa	OdRaCL	Chisq	ErrProb
**"I am affected/hurt by reproaches, criticism, blames"** correlated with:
I refused invitations for fear I would feel lonely	2,48	2,29–2,7	466,53	2×10⁻¹⁰³
When I do something, I become intimidated if someone looks at me	3,85	3,54–4,19	1037,2	1×10⁻²²⁷
Because of timidity, I missed opportunities in my personal or professional life	2,47	2,27–2,68	473,7	5×10⁻¹⁰⁵
I feel uncomfortable in the presence of unknown people.	3,3	3,02–3,6	745,26	4×10⁻¹⁶⁴
**"I feel affected/hurt when insulted, humiliated, demeaned, abased"** correlated with:
When I do something, I become intimidated if someone looks at me	2,73	2,5–2,98	529,57	4×10⁻¹¹⁷

**Table 2:** **Appreciation–desire** correlated with **rumination**

The item to be correlated with	OdRa	OdRaCL	Chisq	ErrProb
**"I am affected/hurt by reproaches, criticism, blames"** correlated with:
It is difficult for me to fall asleep because I am troubled by thoughts	2,56	2,36–2,78	521,21	2×10⁻¹¹⁵
After an argument, I think about what happened and what I should have done or said	4,88	4,44–5,36	1188,5	2×10⁻²⁶⁰
Unpleasant memories persist in my mind	3,62	3,33–3,94	952,05	5×10⁻²⁰⁸
I think of past events	3,29	3–3,6	692,71	1×10⁻¹⁵²
I forget the blunders I’ve made	2,64	2,44–2,87	556,2	6×10⁻¹²³
**"I feel affected/hurt when insulted, humiliated, demeaned, abased"** correlated with:
I make it up with a person that has offended ME	2,87	2,63–3,14	550,98	8×10⁻¹²²
After an argument, I think about what happened and what I should have done or said	3,72	3,39–4,08	822,24	8×10⁻¹⁸¹
After an unpleasant event, I rack my brains trying to figure out how I should have acted	4,29	3,92–4,7	1056,5	1×10⁻²³¹
Unpleasant memories persist in my mind	3,33	3,05–3,64	743,95	8×10⁻¹⁶⁴
**"I want to be appreciated and/or admired" **correlated with:
After an argument, I think about what happened and what I should have done or said	2,85	2,6–3,12	515,52	4×10⁻¹¹⁴
After an unpleasant event, I rack my brains trying to figure out how I should have acted	2,65	2,42–2,9	472,09	1×10⁻¹⁰⁴
**"It bothers me to be unfairly accused and/or disregarded"** correlated with:
After an argument, I think about what happened and what I should have done or said	2,77	2,53–3,03	504,24	1×10⁻¹¹¹
After an unpleasant event, I rack my brains trying to figure out how I should have acted	2,53	2,32–2,76	459,31	7×10⁻¹⁰²

**Table 3:** **Appreciation–desire** correlated with **injustice–dislike**

The item to be correlated with	OdRa	OdRaCL	Chisq	ErrProb
**"I want my achievements to be appreciated"**correlated with:
I am indignant with injustice	3,79	3,47–4,15	897,1	4×10⁻¹⁸⁷
Good manners matter to me	2,96	2,71–3,23	612,39	3×10⁻¹³⁵
**"It affects me when someone credits him/herself with (is credited with/makes a merit of) my achievements"** correlated with:
I am indignant with injustice	4,82	4,41–5,27	1280,9	2×10⁻²⁸⁰
**"It bothers me to be unfairly accused and/or disregarded"** correlated with:
Good manners matter to me	3,41	3,12–3,71	805,34	4×10⁻¹⁷⁷

**Table 4:** **Discontent/worry about couple/partner** correlated with **appreciation–desire**

The item to be correlated with	OdRa	OdRaCL	Chisq	ErrProb
**"I am affected/bothered by my partner’s absence from home"** correlated with:
I am affected/hurt by reproaches, criticism, blames	2,62	2,41–2,85	520,19	4×10⁻¹¹⁵
I want to be appreciated and/or admired	2,85	2,6–3,13	508,78	1×10⁻¹¹²
**"I am hurt/bothered if my partner does not show me affection/attention"** correlated with:
I want to be appreciated and/or admired	4,54	4,15–4,98	1131,6	4×10⁻²⁴⁸
I am affected/hurt by reproaches, criticism, blames	4,19	3,84–4,58	1064,3	2×10⁻²³³
I feel affected/hurt when insulted, humiliated, demeaned, abased	3,75	3,43–4,1	874,04	4×10⁻¹⁸²
It bothers me to be unfairly accused and/or disregarded	3,19	2,92–3,48	701,04	2×10⁻¹⁵⁴
I want my successes to be acknowledged	3,01	2,76–3,29	622,86	2×10⁻¹³⁷

**Table 5:** **Discontent/worry about couple/partner** correlated with **injustice–dislike**

The item to be correlated with	OdRa	OdRaCL	Chisq	ErrProb
**"I would like to make my partner (spouse, friend) behave otherwise"** correlated with:
I consider that in family/couple/friendship relationships, I got what I deserved	2,89	2,66–3,14	650,36	2×10⁻¹⁴³
In family/couple/friendships relationships I have been treated as I deserved.	2,82	2,58–3,08	550,98	8×10⁻¹²²
I consider that in family/couple/friendship relationships, I have been treated fair	2,73	2,5–2,97	531,19	2×10⁻¹¹⁷
**"I like the way my partner (spouse, friend) treats me/talks to me"** correlated with:
I consider that in family/couple/friendship relationships, I got what I deserved	4,62	4,24–5,02	1321,8	2×10⁻²⁸⁸
I consider that in family/couple/friendship relationships, I have been treated fair	4,07	3,73–4,43	1076,7	4×10⁻²³⁶
In family/couple/friendships relationships I have been treated as I deserved.	3,94	3,62–4,3	1013,9	2×10⁻²²²

**Table 6:** **Discontent/worry about couple/partner** correlated with **rumination**

The item to be correlated with	OdRa	OdRaCL	Chisq	ErrProb
**"I am concerned about my relationship"** correlated with:
Unpleasant memories persist in my mind	2,52	2,32–2,73	501,82	4×10⁻¹¹¹
I think of past events	2,44	2,25–2,65	457,08	2×10⁻¹⁰¹

**Table 7:** **Discontent/worry about couple/partner** correlated with **conflict–proneness**

The item to be correlated with	OdRa	OdRaCL	Chisq	ErrProb
**"I am hurt/bothered if my partner does not show me affection/attention"** correlated with:
Conflicts/fights affect/bother me	0,35	0,32–0,38	531,63	1×10⁻¹¹⁷

**Table 8:** **Timidity** correlated with **rumination**

The item to be correlated with	OdRa	OdRaCL	Chisq	ErrProb
**"I refused invitations for fear I would feel lonely"** correlated with:
Unpleasant memories persist in my mind	2,62	2,42–2,85	544,91	2×10⁻¹²⁰
After an unpleasant event, I rack my brains trying to figure out how I should have acted	2,67	2,44–2,92	467,01	1×10⁻¹⁰³
**"When I do something, I become intimidated if someone looks at me"** correlated with:
After an unpleasant event, I rack my brains trying to figure out how I should have acted	3,43	3,15–3,75	797,74	2×10⁻¹⁷⁵
Unpleasant memories persist in my mind	3,01	2,78–3,27	721,22	7×10⁻¹⁵⁸
After an argument, I think about what happened and what I should have done or said	3,06	2,8–3,35	611,09	6×10⁻¹³⁵
I think of past events	2,86	2,62–3,13	576,73	2×10⁻¹²⁷
It is difficult for me to fall asleep because I am troubled by thoughts	2,55	2,35–2,76	523,69	7×10⁻¹¹⁶
My sleep is disturbed by dreams about things that have preoccupied me during the day	2,52	2,32–2,73	513,11	1×10⁻¹¹³
**"Because of timidity, I missed opportunities in my personal or professional life" correlated with**
Unpleasant memories persist in my mind	2,42	2,23–2,63	459,71	6×10⁻¹⁰²
**"I feel uncomfortable in the presence of unknown people."** correlated with:
Unpleasant memories persist in my mind	2,71	2,49–2,95	563,09	2×10⁻¹²⁴
After an unpleasant event, I rack my brains trying to figure out how I should have acted	2,89	2,63–3,18	515,01	5×10⁻¹¹⁴
I think of past events	2,47	2,28–2,69	468,58	7×10⁻¹⁰⁴

**Table 9:** **Emotivity** correlated with **appreciation–desire**

The item to be correlated with	OdRa	OdRaCL	Chisq	ErrProb
**"I am emotional"** correlated with
I am affected/hurt by reproaches, criticism, blames	3,89	3,58–4,23	1050,7	2×10⁻²³⁰
I feel affected/hurt when insulted, humiliated, demeaned, abased	3	2,74–3,27	618,25	2×10⁻¹³⁶

**Table 10:** **Emotivity** correlated with **rumination**

The item to be correlated with	OdRa	OdRaCL	Chisq	ErrProb
**"I get excited/I am moved/I am touched"** correlated with:
After an unpleasant event, I rack my brains trying to figure out how I should have acted	0,36	0,33–0,39	501	6×10⁻¹¹¹
**"I am emotional"** correlated with:
After an unpleasant event, I rack my brains trying to figure out how I should have acted	3,53	3,23–3,86	817,55	8×10⁻¹⁸⁰
I think of past events	2,92	2,68–3,18	623,36	1×10⁻¹³⁷
After an argument, I think about what happened and what I should have done or said	3,11	2,84–3,41	613,2	2×10⁻¹³⁵
Unpleasant memories persist in my mind	2,51	2,31–2,71	512,79	2×10⁻¹¹³

**Table 11:** **Rumination** correlated with **injustice–dislike**

The item to be correlated with	OdRa	OdRaCL	Chisq	ErrProb
**"It is difficult for me to fall asleep because I am troubled by thoughts"** correlated with:
Life has been unfair to me.	2,86	2,64–3,11	641,43	2×10⁻¹⁴¹
I suffered injustice	2,64	2,41–2,89	455,29	5×10⁻¹⁰¹
**"Unpleasant memories persist in my mind"** correlated with:
Life has been unfair to me.	2,92	2,69–3,17	667,75	3×10⁻¹⁴⁷
I suffered injustice	3,27	2,98–3,59	664,09	2×10⁻¹⁴⁶
I was unjustly punished	2,6	2,4–2,82	550,46	1×10⁻¹²¹
I consider that in family/couple/friendship relationships, I have been treated fair	2,63	2,42–2,87	495,87	8×10⁻¹¹⁰
I consider that in family/couple/friendship relationships, I got what I deserved	2,5	2,31–2,72	490,85	9×10⁻¹⁰⁸
**"I think of past events"** correlated with:
I suffered injustice	2,62	2,41–2,86	491,32	7×10⁻¹⁰⁸

**Table 12:** **Rumination** correlated with **conflict–proneness**

The item to be correlated with	OdRa	OdRaCL	Chisq	ErrProb
**"After an argument, I think about what happened and what I should have done or said"** correlated with:
Conflicts/fights affect/bother me	0,26	0,23–0,28	845,94	6×10⁻¹⁸⁶
**"After an unpleasant event, I rack my brains trying to figure out how I should have acted"** correlated with
Conflicts/fights affect/bother me	0,22	0,2–0,25	1057,4	6×10⁻²³²
**"Unpleasant memories persist in my mind"**correlated with:
I get into conflict with close persons (relatives, friends)	2,98	2,72–3,27	571,57	3×10⁻¹²⁶

**Table 13:** **Conflict–proneness** correlated with **emotivity**

The item to be correlated with	OdRa	OdRaCL	Chisq	ErrProb
**"Conflicts/fights affect/bother me"** correlated with:
I get excited/I am moved/I am touched	2,85	2,6–3,14	497,53	3×10⁻¹¹⁰
I am emotional	0,33	0,3–0,36	592,29	8×10⁻¹³¹

**Table 14:** **Conflict–proneness** correlated with **appreciation–desire**

The item to be correlated with	OdRa	OdRaCL	Chisq	ErrProb
**"Conflicts/fights affect/bother me"** correlated with:
I feel affected/hurt when insulted, humiliated, demeaned, abased	0,26	0,24–0,29	854,23	9×10⁻¹⁸⁸
I am affected/hurt by reproaches, criticism, blames	0,2	0,19–0,22	1179,8	2×10⁻²⁵⁸

**Table 15:** **Conflict–proneness** correlated with **injustice–dislike**

The item to be correlated with	OdRa	OdRaCL	Chisq	ErrProb
**"Conflicts/fights affect/bother me"** correlated with:
I am indignant with injustice	0,32	0,29–0,36	534,72	3×10⁻¹¹⁸

## Discussions

Practically all subdomains have scores of very strong inner associations. Many of these associations are between semantically akin items – these are uninteresting from a psychological perspective, but are priceless for testing the consistency of the answers. Others express commonsensical psychological facts, and they may be useful if revealing a certain hierarchy (for example, the couple relationships seem to be mostly affected by insults and humiliations, less so by lack of attention/affection, and least by infidelity). Some subdomains are strongly connected with others: couple/partner with injustice–dislike and relatives/friends; sociability with tendency–to–isolation and timidity; timidity with emotivity; appreciation–desire with timidity, emotivity, and rumination; rumination with conflict-proneness etc. Some of these associations are between items with similar significance, revealing either incorrect item–to–subdomain assignment, or an inclusion relation between a broader, more general item and a narrower, more particular one. We have not presented the corresponding results in the present paper because they were too many (for the limited space available to our paper) and more or less obvious.

Nonetheless, some associations reveal more or less subtle psychological mechanisms, such as: most individuals consider that they do not deserve to be treated badly by other people; timidity and emotivity are associated with the dislike of being slighted; the conflict avoiding inclination is associated with persistent unpleasant memories etc. Although one could have expected that the most important items of this second domain (“Interaction with other people”) should be those expressing the preoccupation with other people (especially with the partner), our analysis revealed that the appreciation desire items have the upper hand, and all the others seem to orbit around them. Hence, we shall start our analysis with these items.

### Appreciation–desire

Timid people seem to be more susceptible to criticism (see **Table 1**). This may suggest that timidity is not a primary PF, but a secondary one, derived from the sensitivity to insults. We could infer that shy individuals are generally easily hurt, as they tend to over- or misinterpret the various attitudes of other people, regarding as reproaches or blames gestures or words which are not so intended. Consequently, social integration could be improved by interventions that diminish the vulnerability to humiliation (by teaching the concerned individuals to correctly evaluate other people’s attitudes) – the yield of such a strategy could be considerable as there are already many psychotherapeutic techniques (cognitive behavioral therapy, transactional analysis etc.) aimed thereto.

In view of the association of timidity with both appreciation desire, and rumination (see below), there is an expected correlation between this last two PFs (see **Table 2**). One can hardly establish which of these is the primary one. They may be both manifestations of one and the same psychic tendency, emanation of an introverted nature. On the other hand, there are appreciation-craving people that are definitely extroverted, and thus less prone to brooding. Such people are very outgoing and interaction-inclined. Consequently, we might speculate that in a given individual the appreciation desire might be modulated by the balance between extroversion /introversion, resulting in a sociable (and possibly more altruistic) nature in extrovert people, and in a timid and brooding nature in introvert people (we shall assess in a future paper whether there is an association between sociability and altruism, as we have assigned the altruism related items to the fourth domain). The practical relevance of these insights may be sizeable and at least three–pronged: (1) psychotherapeutically tackling the appreciation desire may diminish the risk for both altruism associated illnesses and timidity/rumination related ones, (2) timidity/rumination related issues (including internal pathology) could be better understood and approached if one takes into account the appreciation desire as their probable root, (3) at their turn, the undesirable consequences of unbridled sociability/altruism can be more efficiently managed if regarded as deriving from the appreciation desire.

Expectedly, the desire to be appreciated is correlated with injustice-dislike (see **Table 3**), as these PFs may explain one another. Actually, they may be regarded as alternative phrasings of the same psychic tendency. Probably all people consider they deserve to be appreciated (and even more so those outwardly pretending they are worthless!), therefore being appreciated equates to (or at least is included in) being treated fairly.

### Discontent/worry about couple/partner

Offenses are what the majority of people (we should say the majority of women) dislike most from their partners’ behavior. Somehow less important seems to be the lack of affection/attention, which is mostly correlated with the partner’s absence from home (p = 7×10⁻²⁸⁰). Perceiving the relation between these two items in the reverse order, we notice that the partner’s absence from home makes one feel unloved and uncared for; it may also elicit fear of infidelity (p = 4×10⁻²²⁶) and, to a lesser degree, worries about the absent partner (p = 6×10⁻¹⁴³) (results not shown here).

People tend to regard the lack of affection from the partner and the partner’s absence from home as signs of not being appreciated (see **Table 4**), and thus blame themselves in circumstances unrelated to their person. Again, the fundamental issue is probably not the partner’s attitude or schedule, but the individual’s propensity to feel underappreciated. This could have important repercussions from a therapeutic viewpoint: many couple–related problems may be solved through strategies designed with an eye to this information. The partner’s heavy work timetable might be annoying, but this should not be regarded as a sign of lack of respect.

The partner’s bad behavior is generally perceived as undeserved. The corresponding items, although placed in the injustice-dislike subdomain, also strongly refer to couple relationships. Besides, it seems that those discontents with their couple-relationship tend also to be annoyed with the behavior of other close people. Frequently, people discontented/worried about couple/partner have a strong desire to be treated fairly (equivalent to injustice–dislike), especially by their partners (see **Table 5**), which is a predictable correlation, and thus of little consequence. It is difficult to decide whether the desire to be treated fairly by the partner should be in the couple/partner subdomain or in the equity/fairness desire subdomain, as it has correlations of comparable strength with both subdomains.

Expectedly, people concerned with their relationships are more prone to brood upon (unpleasant) events in the past (see **Table 6**), supposedly upon those concerning their relationship. This information might seem trivial if viewed in the sequence “concern about relationship implies more brooding“, but the rumination tendency is probably a more fundamental one, as it is a reflection of the individual’s personality, while the relationship–related worries depend (at least apparently) on exterior events. Therefore, one may wonder whether an inverse sequence might be the correct one: the rumination–prone people tend to have more troubled relationships (or to regard their relationships as such or to be bothered more by minor occurrences etc.). This could be a far less commonplace information, as psychological interventions aimed at positively transforming the rumination tendency might result in improved relationships (and thus decrease the pathology specifically related to troubled–relationships).

The more is one bothered by lack of affection, the more is he/she bothered by conflicts (see **Table 7**), a result that could have been easily anticipated, as conflicts can scarcely be regarded as love tokens.

Somehow amusing are the correlations of the item “I worry for my partner (spouse/friend)”: those worried for their partner’s fate are also striving to figure out better ways of responding to unpleasant events (p = 9×10⁻⁷⁷), have their sleep disturbed by persistent thoughts (p = 4×10⁻⁶⁷), consider that they have suffered injustice (p = 8×10⁻⁶⁴), and feel hurt by demeaning words (p = 3×10⁻⁵⁸) – we could muse about who might be the perpetrator of their partner’s ill–fate.

### The sociability related items

The sociability related items are generally inversely correlated with the timidity related ones, suggesting that these two psychic aspects should be viewed as the opposite poles of a single psychic axis, and therefore the corresponding items should be pooled together in a single subdomain. We haven’t shown here the relevant results because they are too many (85 correlations with a ErrProb <10⁻¹⁰⁰) and too predictable.

### Timidity related items

Besides the aforementioned association between the discomfort felt in the presence of other people and the sensitivity to offenses and criticism, there are strong correlations between timidity and rumination (see **Table 8**), explicable if we regard both PFs as reflections of an internally oriented nature – an introvert person might be expected to both shy external interactions, and dwell upon past events. A timid individual tends to persistently think about how he/she should have acted in a certain situation or might be haunted by the question whether he/she has acted properly in a given circumstance or might regret not having capitalized on an opportunity etc. From a practical point of view, this could have some therapeutic consequences: an intervention designed to improve social interaction might result in decreased inclination to rumination (and possibly to less rumination-associated pathology) or, conversely, a therapy–induced decline in the brooding tendency might improve one’s ability to relate to other people.

### Emotivity related items

Similarly to the timidity related items, emotivity related ones are also associated with the sensitivity to insults (see **Table 9**) and with the tendency to rumination (see **Table 10**). Besides, emotivity is more frequently encountered in people bothered by conflicts (see **Table 13**). An explanation might be again that both emotivity and rumination are common in introverted people. Furthermore, emotional people are expected to have sensitive natures, and some of the things they may be sensitive to are painful memories, insults, and conflicts.

### Rumination related items

There are strong correlations between the items expressing the inclination to rumination and those reflecting the desire to be fairly treated (see **Table 11**) and to be appreciated (see **Table 2**). In the light of the previously unraveled correlation of rumination with appreciation-desire, the association with injustice-dislike seems commonsensical. Among the correlations with the conflict related items (see **Table 12**), the most interesting is with the inclination to get into conflict with close persons. One could argue that it is common for people to dwell on the unpleasant memories left in the wake of a conflict (which of course is true), but the converse might reflect the reality even better – brooding people are both more sensitive, and more prone to conflicts. Attempts to dissipate their conflict–seeking nature are doomed to failure if their rumination propensity is not addressed. Conflict avoiding or solving strategies are met with but limited results as long as the individual continues to live in the past and draws heavily on his/her old resentments

### Conflict–proneness

Appreciation–desire is inversely correlated with conflict–proneness (see **Table 14**) – a further expected correlation as insults and criticism are very often regarded (especially by the sensitive natures) as conflicts.

The more one is bothered by conflicts, the more is he/she averse to injustice (see Table **Table 15**). Interestingly, the item “I try to avoid conflicts/fights” is much less strongly associated with the injustice-indignation (p ≈ 1×10⁻¹⁰), suggesting that the injustice–indignant have a strong emotional reaction to conflicts, but they are not attempting to avoid them. Many of those that declare to be troubled by conflicts are actually interiorly cultivating and perpetuating the conflict.

One explanation for the correlations with the emotivity, the rumination, and the justice–desire subdomains could be that one needs to fight for one’s rights, which a conflict–annoyed individual would be inclined to avoid, therefore inviting injustice – consequently, all that is left to him is to get indignant.

## Conclusions

Insults and humiliations seem to be the most important concern in couple relationships, while the relationship–related dissatisfaction is associated with a sense on unfairness. However, the core PF of the Interaction–with–other–people domain is the desire to be appreciated (and the corresponding dread of humiliations and criticism). This is probably one of the main determinants of the course of interpersonal relationships and probably of the psychoemotional predisposition to disease. It may be modulated by the individual’s inner or outer oriented nature. In introverted persons it could engender PFs such as timidity and rumination–proneness and/or it may nurture relational discontent. Our suggestion is that any intervention for disorders psychoemotionally grounded in troubled relationships should have the sense of being unappreciated as one of the central targets.
